# Efficacy of cupping therapy on pain outcomes: an evidence-mapping study

**DOI:** 10.3389/fneur.2023.1266712

**Published:** 2023-10-26

**Authors:** Liaoyao Wang, Ziling Cai, Xuanlin Li, Aisong Zhu

**Affiliations:** ^1^School of Basic Medical Sciences, Zhejiang Chinese Medical University, Hangzhou, China; ^2^Key Laboratory of Blood-Stasis-Toxin Syndrome of Zhejiang Province, Hangzhou, China; ^3^Zhejiang Engineering Research Center for “Preventive Treatment” Smart Health of Traditional Chinese Medicine, Hangzhou, China

**Keywords:** cupping therapy, pain-related conditions, systematic review, meta-analysis, evidence mapping

## Abstract

**Objective:**

Cupping therapy is an ancient technique of healing used to treat a variety of ailments. An evidence-mapping study was conducted to summarize the existing evidence of cupping therapy for pain-related outcomes and indicate the effect and the quality of evidence to provide a comprehensive view of what is known.

**Methods:**

PubMed, Cochrane Library, Embase, and Web of Science were searched to collect the meta-analyses investigating the association between cupping therapy and pain-related outcomes. The methodological quality was assessed by using the AMSTAR 2 tool. Significant outcomes (*p* < 0.05) were assessed using the GRADE system. The summary of evidence is presented by bubble plots and human evidence mapping.

**Results:**

Fourteen meta-analyses covering five distinct pain-related conditions were identified and assessed for methodological quality using the AMSTAR 2, which categorized the quality as critically low (36%), low (50.0%), moderate (7%), and high (7%). In accordance with the GRADE system, no high-quality evidence was found that demonstrates the efficacy of cupping therapy for pain-related outcomes. Specifically, for neck pain, there were two moderate-quality, four low-quality, and two very low-quality evidence, while only one very low-quality evidence supports its efficacy in treating herpes zoster and one low-quality evidence for chronic back pain. Additionally, for low back pain, there were two moderate-quality, one low-quality, and four very low-quality evidence, and for knee osteoarthritis, three moderate-quality evidence suggest that cupping therapy may alleviate pain score.

**Conclusion:**

The available evidence of very low-to-moderate quality suggests that cupping therapy is effective in managing chronic pain, knee osteoarthritis, low back pain, neck pain, chronic back pain, and herpes zoster. Moreover, it represents a promising, safe, and effective non-pharmacological therapy that warrants wider application and promotion.

**Systematic review registration**: https://www.crd.york.ac.uk/prospero/display_record.php?ID=CRD42021255879, identifier: CRD42021255879.

## 1. Introduction

The definition of pain has been revised to an unpleasant sensory and emotional experience associated with, or resembling that associated with, actual or potential tissue damage according to the International Association for the Study of Pain (IASP) (1). Pain is the principal reason why individuals seek medical care, with three of the top ten reasons being osteoarthritis, back pain, and headaches (2). Chronic pain poses a significant personal and economic burden, affecting over 30% of the global population and causing psychological distress and sleep issues (3). The Global Burden of Diseases study identified low back pain and migraine as two of the five leading causes of years lived with disability (YLDs) (4). In China, the annual total treatment cost of chronic pain may surpass 500 billion yuan (approximately 685 billion dollars) (5). Although many medications may have limited effectiveness, they often come with significant side effects that can be compounded (6). As a result, alternative complementary treatments are increasingly crucial in the management of pain-related conditions. The demand for complementary and integrative medicine approaches has been on the rise, including mind–body interventions, acupuncture therapy, and other traditional Chinese medicine (TCM) practices (7). Among these, cupping therapy stands out due to its simplicity, safety, and efficacy. Cupping therapy has been widely used in various fields of medicine, including internal medicine, external medicine, gynecology, pediatrics, and particularly in conditions related to pain, skin diseases, knee osteoarthritis, migraines, and other ailments (8).

Cupping therapy, an ancient healing modality, has long been a mainstay in TCM, as well as being documented in the historical records of other regions, including ancient Egypt, Greece, and India. This valuable therapeutic technique has been utilized for thousands of years, and its benefits have been recognized and applied worldwide. It is not only a part of TCM but also recorded in ancient Egypt, Greece, India, and other regions. It is a precious asset to people and has been used worldwide. Cupping therapy involves the application of cups to targeted acupoints or specific skin regions, which creates a negative pressure (9, 10). The modalities of cupping can be broadly classified into dry cupping, wet cupping, massage cupping, etc. (11).

Cupping therapy, initially used as a pain relief method, has now been extended to a broad range of medical conditions (12). Recent evidence shows that this therapy may offer potential benefits for a variety of conditions such as myofascial pain (13), low back pain, ankylosing spondylitis, knee osteoarthritis, neck pain, herpes zoster, migraine, plaque psoriasis, and chronic urticaria (14). For pain-related conditions, cupping might be used as a useful intervention because it decreases the pain level and improves blood flow to the affected area with low adverse effects (15). A clinical study has confirmed that cupping was more effective in improving pain and functional disability in people with persistent non-specific low back pain when compared to sham therapy (16). A single session of dry cupping therapy may be an effective short-term treatment method for immediately reducing pain (17). However, it is worth noting that there exist clinical research findings that do not align with this conclusion. Cupping therapy was not superior to sham cupping for improving pain, physical function, mobility, quality of life, psychological symptoms, or medication use in people with non-specific chronic low back pain (18). Despite this, the National Center for Complementary and Integrative Health (NCCIH) (U.S.) states that although cupping therapy may have some effect in reducing pain, the available evidence is currently insufficient (19). Moreover, although research on this form of therapy has increased, there remains a lack of comprehensive surveys that summarize the efficacy of cupping therapy in managing pain-related conditions. To bridge this gap, the present study endeavors to furnish a comprehensive evaluation of related meta-analyses pertaining to cupping therapy, with a particular focus on the outcomes of pain, as well as to carry out evidence mapping. The primary aim of this study was to provide insights for forthcoming research endeavors.

## 2. Methods

### 2.1. Protocol and registration

The protocol of this evidence mapping was registered at the PROSPERO (CRD 42021255879).

### 2.2. Data sources and search strategy

PubMed, Cochrane Library, Embase, and Web of Science were searched to identify the systematic reviews with meta-analyses on the relationship between cupping therapy and any pain-related conditions published from inception until 15 April 2023. Medical Subject Heading (MeSH) terms and their variants were used for the search strategy for the following terms: “cupping therapy,” “cupping,” “cupping treatment,” “meta-analysis,” “meta-analysis as topic,” “systematic review.” Only published articles in English were considered. We also conducted an extensive review of the pertinent literature, encompassing a range of narrative synopses within the domain. Such a comprehensive approach allowed us to ensure that no substantive sources were overlooked. The details of the search strategies for all databases are given in Supplementary Table S1.

### 2.3. Inclusion and exclusion criteria

All systematic reviews with meta-analysis related to cupping therapy (including but not limited to dry cupping, wet cupping, moving cupping, etc.) for any pain-related outcomes were included. Conference abstracts, letters, protocols, overviews, and systematic reviews without quantitative meta-analysis were excluded.

### 2.4. Study selection

All records identified from four databases were imported into Endnote X9 software, and duplicate records removed before screening. After eliminating duplicates, two authors (LW and XL) independently read the titles, abstracts, or full text until all studies are confirmed. Ambiguity was resolved by group discussion.

### 2.5. Data extraction

For each eligible study, two authors (LW and ZC) extracted the following data independently: first author, publication year, country, study type, type of disease or disorder, number of studies, sample size, intervention, comparison, outcome, type of metric with 95% confidence interval [CI] (i.e., odds ratio [OR], relative risk [RR], risk ratio [HR], standardized mean deviation [SMD], mean deviation [MD]), adverse effect, and the main findings. Ambiguity was resolved by group discussion with the other author (AZ).

### 2.6. Methodological quality assessment

A measurement tool to assess systematic reviews 2 (AMSTAR 2) (20), which contains 16 items, was used to assess the methodological quality and ranks the quality from critical low to high of the included studies. Two researchers (LW and ZC) independently evaluated the quality of the included studies. The items of the AMSTAR 2 checklist are shown in Supplementary Table S2.

### 2.7. Evidence quality assessment

We used Grading of Recommendations, Assessment, Development, and Evaluation (GRADE) (21) to assess the quality of evidence for each outcome on four degrees (high, moderate, low, and very low quality) by two reviewers (LW and XL) independently. Any disagreement between reviewers was resolved by discussion, and consistent results were reached finally.

### 2.8. Evidence mapping presentation

The evidence mapping findings were depicted using bubble plots in a graphical form. Each bubble in the chart denotes clinical evidence from studies that explored the efficacy of cupping for specific pain-related conditions and clinical indications. Excel 2021 was utilized to design the evidence mapping. The X-axis indicates the effect size of the primary outcome visual analog scale (VAS) (*p* < 0.05), while the Y-axis denotes the number of articles. The size of the bubbles corresponds to the total population’s sample size for the effects of cupping, with bigger bubbles representing a larger sample size. The colors symbolize the different interventions of cupping and non-cupping groups. In addition, we aimed to summarize all the distinct qualities of evidence relating to different pain conditions in human evidence mapping.

## 3. Results

### 3.1. Study selection

**Figure 1:**
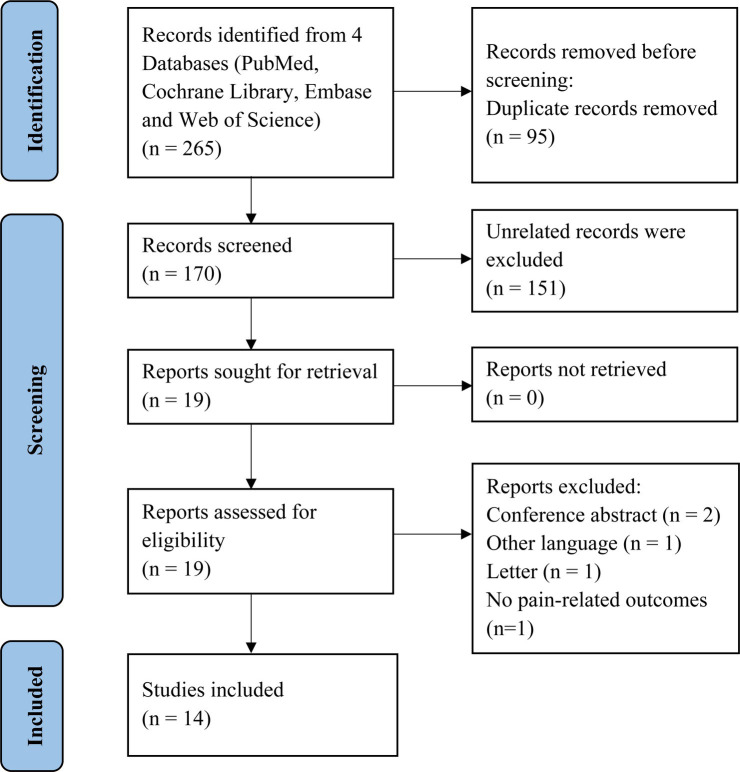
Flow chart of the literature search and screening process.

A total of 265 records were identified. After removing duplicates and screening the titles and abstracts, there were 19 potentially eligible studies. We finally included 14 studies (22–35) after assessing for eligibility including five types of pain-related conditions. The study selection process is shown in Figure 1.

### 3.2. Study characteristics

Five studies (23, 26, 28, 30, 31) contained less than ten original studies, while twelve (85.7%) studies (23–30, 32–35) had a combined total sample of over 500 participants. The majority of studies, eight (57.1%) in total (23, 26, 29–31, 33–35), were conducted in China, followed by two (25, 28) in Korea, and one each in Germany (24), Brazil (27), Australia (32), and Iran (22). The included studies investigated various conditions, including low back pain (*n* = 4), neck pain (*n* = 3), knee osteoarthritis (*n* = 2), chronic back pain (*n* = 1), migraine (*n* = 1), chronic pain (*n* = 1), pain-related conditions (*n* = 1), musculoskeletal pain (*n* = 1), and herpes zoster (*n* = 1). The characteristics of included studies are shown in Table 1.

**Table 1:** Characteristics of included studies.

Study	Country	Clinical condition	No. of databases searched	No. of studies	No. of participants	Outcome	Tool for risk of bias assessment
Cramer et al. (24)	Germany	Chronic pain	3	18	1,172	Pain intensity	ROB tool
Moura et al. (27)	Brazil	Chronic back pain	7	16	1,049	Pain intensity score	Jadad scale
Wang 2018 (30)	China	Knee osteoarthritis	7	5	535	VAS, WOMAC-pain	ROB tool
Seo et al. (28)	Korea	Migraine	8	6	510	VAS	ROB tool
Li et al. (26)	China	Knee osteoarthritis	7	7	661	VAS, WOMAC-pain	ROB tool
Zhang et al. (35)	China	Pain-related conditions	6	23	2,845	VAS	ROB tool
Wood et al. (32)	Australia	Musculoskeletal pain	7	21	1,049	VAS, NRS, SMPQ, PPT	Downs & Black (D&B) quality assessment scale
Kim et al. (25)	Korea	Neck pain	9	18	1,683	VAS, NPQ	ROB tool
Wang et al. (31)	China	Low back pain	3	6	458	VAS, MPPI	Jadad scale
Azizkhani et al. (22)	Iran	Non-specific neck pain	10	10	441	VAS	ROB tool
Cao et al. (23)	China	Herpes zoster	5	8	651	Number of patients with PHN after treatment	Agency for Healthcare Research and Quality
Yuan et al. (34)	China	Neck Pain and Low Back Pain	7	11	666	VAS	Cochrane Back Review Group
Shen et al. (29)	China	Low Back Pain	5	10	690	VAS, PPI	ROB tool
Xie et al. (33)	China	Non-specific low back pain	7	13	1,088	VAS, NRS	ROB tool

MPPI, McGill present pain index; NPQ, Northwick Park Neck Pain Questionnaire; NRS, numerical rating scale; PHN, postherpetic neuralgia; PPI, present pain intensity; PPT, pain pressure threshold; ROB, risk of bias; SMPQ, Short Form McGill Pain Questionnaire; VAS, visual analog scale; WOMAC, Western Ontario and McMaster Universities Osteoarthritis Index.

### 3.3. Methodology quality

**Figure 2:**
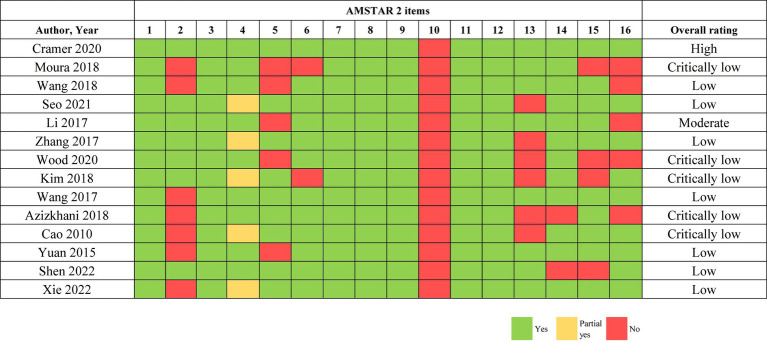
AMSTAR 2 quality assessment.

One high-quality study (24) and one moderate-quality study (26) were included. However, five studies (22, 23, 25, 27, 32) were rated as critically low quality, and seven studies (28–31, 33–35) were rated as low quality. The primary reasons for these downgraded ratings were noted as the absence of registration and protocols (item 2), poor information regarding the source of funding for the original studies in the systematic review and meta-analysis (item 10), and an inadequate explanation of the risk of bias when discussing the results of the review (item 13). The assessment of methodology quality of included studies by AMSTAR 2 is presented in Figure 2.

### 3.4. Evidence quality

Among the 21 outcomes, the quality of evidence was rated as moderate quality, low quality, and very low quality. Eight moderate-quality evidence are for knee osteoarthritis (*n* = 3), neck pain (*n* = 2), low back pain (*n* = 2), and chronic pain (*n* = 1). Six low-quality evidence are for neck pain (*n* = 4), chronic back pain (*n* = 1), and low back pain (*n* = 1). Seven very low-quality evidence are for low back pain (*n* = 4), neck pain (*n* = 2), and herpes zoster (*n* = 1). On the basis of two moderate-quality, four low-quality, and two very low-quality evidence, cupping therapy is found to be effective for treating neck pain. Similarly, two moderate-quality, one low-quality, and four very low-quality evidence support the efficacy of cupping therapy in alleviating low back pain. Additionally, three moderate-quality evidence indicate that cupping therapy is useful in managing knee osteoarthritis, while only one very low-quality evidence supports its efficacy in treating herpes zoster and one low-quality evidence for chronic back pain. Furthermore, one moderate-quality evidence supports the use of cupping therapy in managing chronic pain. Table 2 and Supplementary Table S3 provide a detailed account of the GRADE assessment.

**Table 2:** Evidence quality of cupping therapy and pain-related outcomes.

Study	Condition	NO. of studies	NO. of participants	Outcome	Metrics	ES (95%) CI	I^2^ (%)	Comparison	Evidence quality
Cramer et al. (24)	Chronic Pain	13	718	Pain intensity	SMD	−1.03 [−1.41, −0.65]	81	Cupping vs. no treatment	⨁⨁⨁〇Moderate
Moura et al. (27)	Chronic back pain	10	595	Pain intensity score	AD	−1.59 [−2.07, −1.10]	67.7	Cupping therapy compared to one or more of the following groups: sham, active treatment, waiting list, standard medical treatment, or no treatment	⨁⨁〇〇Low
Wang et al. (30)	Knee osteoarthritis	2	211	VAS	MD	−1.79 [−2.40, −1.18]	0	Dry cupping therapy + Western medicine vs. Western medicine	⨁⨁⨁〇Moderate
		2	211	WOMAC- Pain	MD	−0.73 [−1.03, −0.43]	0	Dry cupping therapy + Western medicine vs. Western medicine	⨁⨁⨁〇Moderate
Li et al. (26)	Knee osteoarthritis	2	211	WOMAC- pain	MD	−1.10 [−1.61, −0.41]	0	Dry cupping therapy + Western medicine vs. Western medicine	⨁⨁⨁〇Moderate
Wood et al. (32)	Musculoskeletal pain (non-specific neck pain)	5	239	VAS	MD	−1.29 [−2.05, −0.53]	94	Dry cupping vs. no treatment	⨁〇〇〇Very low
		4	191	PPT	SMD	−0.40 [−0.69, −0.11]	0	Dry cupping vs. no treatment	⨁⨁〇〇Low
	Musculoskeletal pain (low back pain)	2	196	VAS	MD	−19.38 [−28.09, −10.66]	59	Dry cupping vs. comparative or control group	⨁〇〇〇Very low
		2	160	SMPQ	MD	−11.20 [−13.76, −8.64]	76	Dry cupping vs. comparative or control group	⨁〇〇〇Very low
Kim et al. (25)	Neck pain	5	241	VAS	MD	−2.42 [−3.98, −0.86]	93	Cupping vs. no treatment	⨁〇〇〇Very low
		9	870	VAS	MD	−0.89 [−1.42, −0.37]	88	Cupping vs. active control	⨁⨁〇〇Low
		1	95	NPQ	MD	3.59 [2.02, 5.16]	/	Cupping vs. active control	⨁⨁〇〇Low
		5	534	VAS	MD	−0.87 [−1.14, −0.61]	19	Cupping + active control vs. active control	⨁⨁⨁〇Moderate
Wang et al. (31)	Low back pain	4	280	VAS	SMD	−0.73 [−1.42, −0.04]	87	Cupping vs. medication or usual care	⨁〇〇〇Very low
Azizkhani et al. (22)	non-specific neck pain	5	282	VAS	MD	−0.84 [−1.22, −0.46]	54.7	Cupping therapy vs. other or no treatment	⨁⨁〇〇Low
Cao et al. (23)	Herpes zoster	3	326	Number of patients with PHN after treatment	RR	0.11 [0.02, 0.56]	0	Wet cupping versus medications	⨁〇〇〇Very low
Yuan et al. (34)	Chronic neck pain	2	93	VAS	WMD	−19.0 [−27.61, −10.58]	0	Cupping vs. waitlist	⨁⨁⨁〇Moderate
	Chronic low back pain	7	430	VAS	WMD	−0.54 [−0.89, −0.19]	84.5	Cupping vs. medications	⨁⨁⨁〇Moderate
Shen et al. (29)	Low Back Pain	3	146	VAS	MD	−1.54 [−1.81, −1.26]	0	et cupping vs. non-cupping group	⨁⨁〇〇Low
		4	275	PPI	MD	−2.22 [−3.92, −0.52]	97	wet cupping vs. non-cupping group	⨁〇〇〇Very low
Xie et al. (33)	Non-specific low back pain	9	736	VAS	MD	−1.43 [−2.31, −0.54]	95	blood pricking and cupping vs. other treatments	⨁⨁⨁〇Moderate

AD, absolute difference; CI, confidence interval; ES, effect size; MD, mean deviation; MPPI, McGill present pain index; NPQ, Northwick Park Neck Pain Questionnaire; NRS, numerical rating scale; PHN, postherpetic neuralgia; PPI, present pain intensity; PPT, pain pressure threshold; RR, risk ratio; SMD, standard mean deviation; SMPQ, Short Form McGill Pain Questionnaire; VAS, visual analog scale; WOMAC, Western Ontario and McMaster Universities Osteoarthritis Index.

### 3.5. Evidence mapping

**Figure 3:**
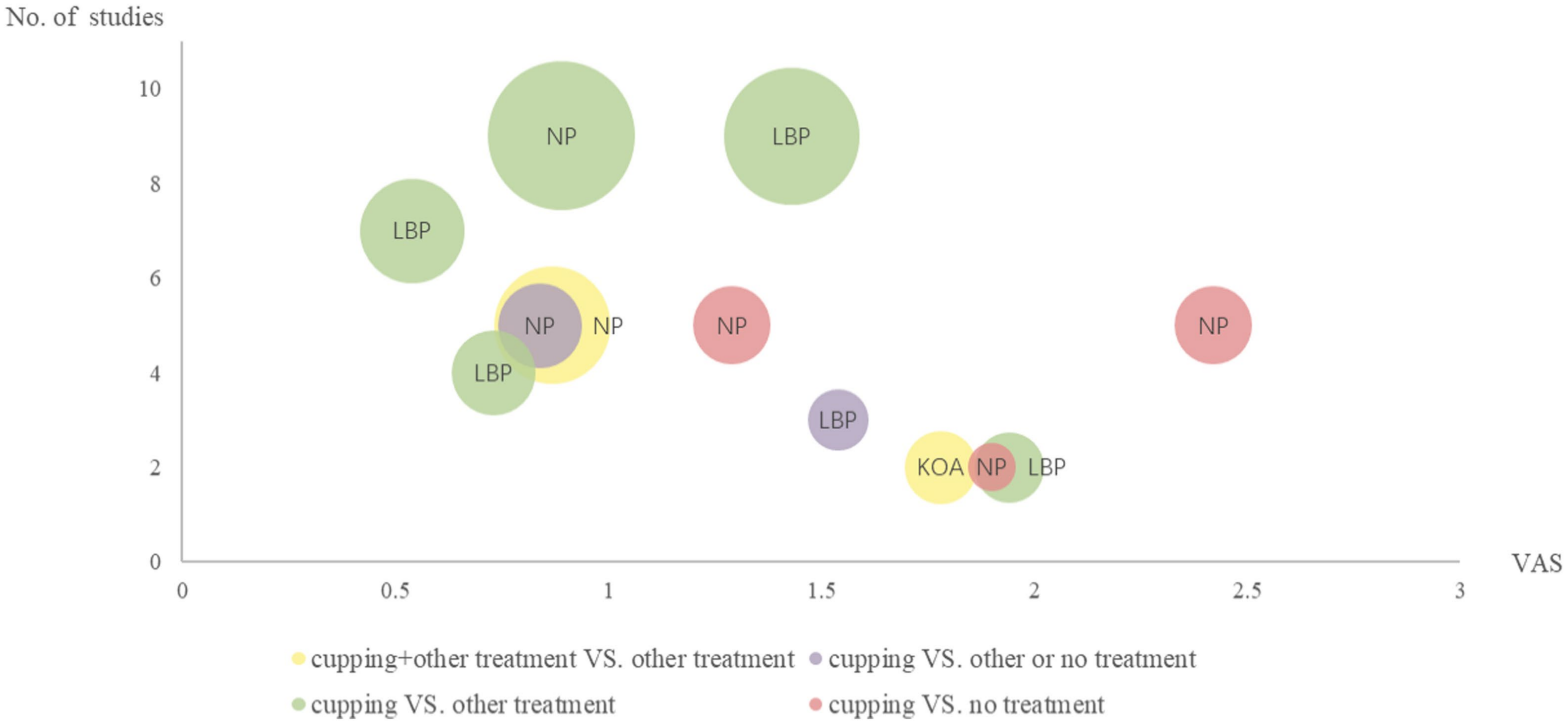
Evidence mapping of cupping therapy. KOA, knee osteoarthritis; NP, neck pain; LBP, low back pain; VAS, visual analog scale. The size of the bubbles corresponds to the total population’s sample size for the effects of cupping, with bigger bubbles representing a larger sample size. The colors symbolize the different interventions of cupping and non-cupping groups.

Figure 3 displays the outcomes of the evidence mapping, which graphically presents the evidence in the form of bubbles. The findings of the evidence mapping revealed that cupping therapy effectively alleviates pain (measured via VAS scores) for neck pain, low back pain, and knee osteoarthritis.

**Figure 4:**
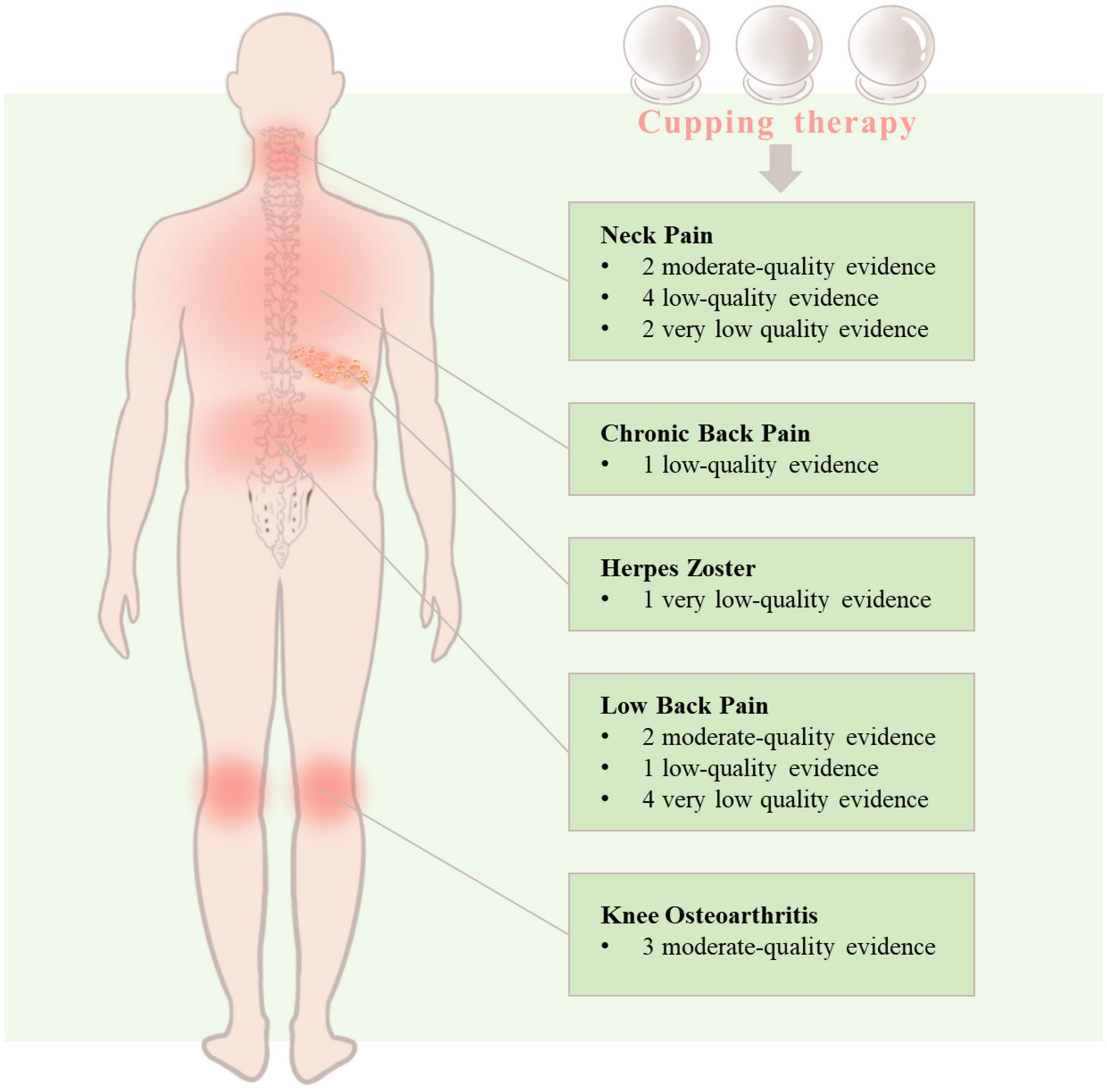
Grade distribution of evidence of cupping therapy for pain-related conditions.

Figure 4 further elucidates the evidence quality for specific pain conditions. For neck pain, there exist two moderate-quality, four low-quality, and two very low-quality evidence. There exists one low-quality evidence for chronic back pain and one very low-quality evidence for herpes zoster that demonstrates cupping therapy’s effectiveness. Moreover, there are two moderate-quality, one low-quality, and four very low-quality evidence for low back pain, while for knee osteoarthritis, three moderate-quality evidence indicate that cupping therapy can alleviate osteoarthritis pain.

## 4. Discussion

### 4.1. Main findings

In the present evidence-mapping study, comprising 14 meta-analyses, the findings highlight the effectiveness of cupping therapy for various pain-related conditions. This comprehensive overview of systematic reviews summarizes the evidence on the efficacy of cupping therapy for several different pain conditions (chronic back pain, knee osteoarthritis, low back pain, neck pain, and herpes zoster). However, none of the meta-analyses provided high-quality evidence on the effectiveness of cupping therapy on pain-related outcomes. In addition, this study shows that cupping therapy is supported by moderate-quality evidence in the relief of a wide range of pain conditions, including chronic pain, knee osteoarthritis pain, low back pain, and neck pain. Some very low to low-quality evidence supports that cupping therapy for chronic back pain, low back pain, neck pain, and herpes zoster. The quality of current evidence provides good support for the clinical use of cupping therapy and direction for future cupping therapy to play a role in the treatment of other pain outcomes.

### 4.2. Potential mechanism of cupping therapy

Cupping therapy is an integral part of TCM, in which its effectiveness is increasingly recognized and substantiated by modern clinical medicine, but its physiological mechanisms have no consensus. The mechanisms of cupping therapy that have been proposed include the promotion of blood circulation, neurological reflex, the gate theory of pain, inflammation-immune reaction, and skin tension increase (36).

#### 4.2.1. Neural

There is converging evidence that cupping therapy can induce comfort and relaxation on a systemic level, and the resulting increase in endogenous opioid production in the brain leads to improved pain control (37). Furthermore, cupping therapy has been found to increase immediate pressure pain thresholds in certain areas (38). In addition, the study has revealed that wet cupping therapy can decrease pain in rats *via* the upregulation of heat shock protein 70 (HSP70) and β-endorphin expression (39).

#### 4.2.2. Hematological

Studies have demonstrated that cupping therapy can augment blood volume and tissue oxygenation at the affected site, whereas reductions in those parameters were observed in the surrounding tissue (40). Moreover, the drawing force in cupping may bring about alteration in blood flow dynamics along with the variation in dermal vascular arrangement. Cupping could positively affect erythrocyte diapedesis from superficial dermal venules. The extravasated erythrocyte may play a mediating role in the proteolytic degradation cascade of hemoglobin. The hemoglobin-derived hemorphins engage opioid receptor signaling and induce the local analgesic effect of cupping (41).

#### 4.2.3. Immune

A study suggests that the mechanism of cupping therapy is that cupping regulates local immunomodulation. The microenvironment is changed when stimulating the surface of the skin, and physical signals transform into biological signals, which also interact with each other in the body. These signaling cascades activate the neuroendocrine-immune system, which produces the therapeutic effect (42).

The mechanisms of cupping therapy for pain reduction are closely related to pain gate theory, diffuse noxious inhibitory controls theory, and reflex zone theory. In summary, several theories have been proposed to explain the effects produced by cupping therapy, and these theories may overlap or alternate, producing various therapeutic effects in a specific disease (43).

### 4.3. Strengths and limitations

To sum up, our study presents the inaugural overview and evidence mapping of cupping therapy for pain-related outcomes, which comprehensively summarizes the extant evidence. The available evidence quality for the effectiveness of cupping therapy ranges from very low to moderate, with an absence of high-quality evidence. Future research endeavors should concentrate on elucidating the underlying mechanisms of cupping therapy, prioritizing avoidance of adverse events, and optimizing the design and execution of clinical investigations.

Unlike the protocol, our study was limited to pain-related outcomes. The rest of the methods and steps basically followed the contents of the protocol. First of all, the increase of the literature of cupping therapy for pain makes it possible to conduct an evidence mapping. Furthermore, there is consensus among authors that in registration, outcomes cover diverse diseases, but focusing on a certain area after literature screening could better reduce bias. Therefore, we proceeded with the evaluation of pain-related outcomes. We utilized stringent inclusion criteria and restricted our review to English-language literature, which may have increased the likelihood of missing relevant studies. Furthermore, we focused solely on pain-related outcomes, potentially neglecting the impact of cupping therapy on other symptoms, such as functional activities. It is worth noting that non-specific and chronic neck pain were included in our analysis under the umbrella term “neck pain,” while non-specific and chronic low back pain were considered collectively as “low back pain.” Nonetheless, it is essential for better-quality research to validate the current evidence.

## 5. Conclusion

Cupping therapy appears to be a promising treatment modality for various pain-related disorders. It is effective in the treatment of chronic pain, knee osteoarthritis, low back pain, neck pain, chronic back pain, and herpes zoster. However, the quality of the evidence supporting these outcomes is mostly low quality, with moderate-quality evidence still less available and no high-quality. Therefore, to strengthen these findings, more high-quality clinical studies are also needed to obtain a higher level of evidence. Nonetheless, the potential benefits of cupping therapy in clinical practice make it a valuable intervention for further research and implementation.

## Data availability statement

The original contributions presented in the study are included in the article/Supplementary material, further inquiries can be directed to the corresponding author.

## Author contributions

LW: Software, Writing – original draft, Writing – review & editing, Conceptualization, Data curation, Formal analysis, Investigation, Methodology. ZC: Data curation, Formal analysis, Investigation, Methodology, Software, Writing – review & editing. XL: Data curation, Formal analysis, Investigation, Methodology, Software, Writing – review & editing. AZ: Conceptualization, Investigation, Supervision, Writing – review & editing.
