# Supplementary material for: Efficacy of cupping therapy on pain outcomes: an evidence-mapping study
Source: Front Neurol. 2023 Oct 26;14:1266712. doi: 10.3389/fneur.2023.1266712 (PMC10640990; doi:10.3389/fneur.2023.1266712)
Supplement: Supplementary file 1 [file Table_1.DOCX]

Supplementary Material

# Supplement 1. Search strategy

(1) PubMed

| **Search** | **Query** | **Items** |
| --- | --- | --- |
| #1 | Search: "Cupping Therapy"[Mesh] Sort by: Most Recent | 85 |
| #2 | Search: ((Cupping Therapy[Title/Abstract]) OR (Cupping[Title/Abstract])) OR (Cupping treatment[Title/Abstract]) | 2239 |
| #3 | #1 OR #2 | 2315 |
| #4 | Search: "Meta-Analysis" [Publication Type] OR "Meta-Analysis as Topic"[Mesh] Sort by: Most Recent | 186298 |
| #5 | Search: (Meta analys*[Title/Abstract]) OR (Systematic review*[Title/Abstract]) | 371012 |
| #6 | #4 OR #5 | 401021 |
| #7 | #3 AND #6 | 71 |

(2) Embase

| **Search** | **Query** | **Items** |
| --- | --- | --- |
| #1 | 'meta analysis (topic)'/exp | 52301 |
| #2 | 'systematic review (topic)'/exp | 30169 |
| #3 | 'meta analysis'/exp | 256692 |
| #4 | #1 OR #2 OR#3 | 323560 |
| #5 | 'cupping therapy'/exp | 498 |
| #6 | cupping:ab,ti OR 'cupping therapy':ab,ti OR 'cupping treatment':ab,ti | 2996 |
| #7 | #5 OR#6 | 3023 |
| #8 | #4 AND #7 | 89 |

(3) Cochrane Library

| **Search** | **Query** | **Items** |
| --- | --- | --- |
| #1 | ("Meta analys*"):ti,ab,kw OR ("Systematic review*"):ti,ab,kw | 12963 |
| #2 | MeSH descriptor: [Meta-Analysis as Topic] explode all trees | 369 |
| #3 | MeSH descriptor: [Systematic Review as Topic] explode all trees | 38 |
| #4 | #1 or #2 or #3 | 14235 |
| #5 | (Cupping):ti,ab,kw OR ("Cupping Therapy"):ti,ab,kw OR ("Cupping treatment"):ti,ab,kw | 3369 |
| #6 | MeSH descriptor: [Cupping Therapy] explode all trees | 18 |
| #7 | #5 or #6 | 3587 |
| #8 | #4 and #7 | 29 |

(4) Web of Science

| **Search** | **Query** | **Items** |
| --- | --- | --- |
| #1 | TI=("Meta analys*") OR TI=("Systematic review*") | 325176 |
| #2 | TI=("Cupping Therapy") OR TI=(Cupping) OR TI=("Cupping treatment") | 12764 |
| #3 | #1 and #2 | 76 |

# Supplement 2. Items of AMSTAR 2

| No. | Item |
| --- | --- |
| 1 | Did the research questions and inclusion criteria for the review include the components of PICO? |
| 2 | Did the report of the review contain an explicit statement that the review methods were established prior to the conduct of the review and did the report justify any significant deviations from the protocol? |
| 3 | Did the review authors explain their selection of the study designs for inclusion in the review? |
| 4 | Did the review authors use a comprehensive literature search strategy? |
| 5 | Did the review authors perform study selection in duplicate? |
| 6 | Did the review authors perform data extraction in duplicate? |
| 7 | Did the review authors provide a list of excluded studies and justify the exclusions? |
| 8 | Did the review authors describe the included studies in adequate detail? |
| 9 | Did the review authors use a satisfactory technique for assessing the risk of bias (RoB) in individual studies that were included in the review? |
| 10 | Did the review authors report on the sources of funding for the studies included in the review? |
| 11 | If meta-analysis was performed, did the review authors use appropriate methods for statistical combination of results? |
| 12 | If meta-analysis was performed, did the review authors assess the potential impact of RoB in individual studies on the results of the meta-analysis or other evidence synthesis? |
| 13 | Did the review authors account for RoB in primary studies when interpreting/discussing the results of the review? |
| 14 | Did the review authors provide a satisfactory explanation for, and discussion of, any heterogeneity observed in the results of the review? |
| 15 | If they performed quantitative synthesis did the review authors carry out an adequate investigation of publication bias (small study bias) and discuss its likely impact on the results of the review? |
| 16 | Did the review authors report any potential sources of conflict of interest, including any funding they received for conducting the review? |

# Supplement 3. Detail of evidence quality of pain-related outcomes

| **Study** | **Disease** | **Intervention** | **Outcomes** | **GRADE** | | | | | **Evidence quality** |
| --- | --- | --- | --- | --- | --- | --- | --- | --- | --- |
|  |  |  |  | **Risk of bias** | **Inconsistency** | **Indirectness** | **Imprecision** | **Publication bias** |  |
| Cramer 2020 | Chronic pain | cupping VS no treatment | Pain intensity | Not serious | Serious | Not serious | Not serious | None | ⨁⨁⨁〇Moderate |
| Moura 2018 | Chronic back pain | Cupping Therapy VS. one or more of the following groups: sham, active treatment, waiting list, standard medical treatment, or no treatment | Pain intensive score | Serious | Serious | Not serious | Not serious | None | ⨁⨁〇〇Low |
| Wang 2018 | Knee Osteoarthritis | Dry cupping therapy + western medicine VS. western medicine | VAS scores | Not serious | Not serious | Not serious | Serious | None | ⨁⨁⨁〇Moderate |
|  |  |  | WOMAC scores - Pain | Not serious | Not serious | Not serious | Serious | None | ⨁⨁⨁〇Moderate |
| Li 2017 | Knee osteoarthritis | Dry cupping therapy + Western medicine VS. western medicine | WOMAC - pain | Serious | Not serious | Not serious | Serious | None | ⨁⨁〇〇Low |
| Wood 2020 | Musculoskeletal pain and range of motion (Non-specific neck pain) | Dry cupping VS. no treatment | VAS | Serious | Serious | Not serious | Serious | None | ⨁〇〇〇Very low |
|  |  |  | PPT | Serious | Not serious | Not serious | Serious | None | ⨁⨁〇〇Low |
|  | Musculoskeletal pain and range of motion (Low back pain) | Dry cupping vs. comparative or control group | VAS | Serious | Serious | Not serious | Serious | None | ⨁〇〇〇Very low |
|  |  |  | SMPQ | Serious | Serious | Not serious | Serious | None | ⨁〇〇〇Very low |
| Wang 2017 | Low back pain | Cupping VS. medication or usual care | VAS | Serious | Serious | Not serious | Serious | None | ⨁〇〇〇Very low |
| Azizkhani 2018 | non-specific neck pain | Cupping therapy VS. other or no treatment | VAS | Not serious | Serious | Not serious | Serious | None | ⨁⨁〇〇Low |
| Kim 2018 | Neck pain | Cupping VS. active control | VAS | Serious | Serious | Not serious | Not serious | None | ⨁⨁〇〇Low |
|  |  |  | NPQ | Serious | Not applicable | Not serious | Serious | None | ⨁⨁〇〇Low |
|  |  | Cupping + active control VS. active control | VAS | Serious | Not serious | Not serious | Not serious | None | ⨁⨁⨁〇Moderate |
|  |  | Cupping VS. no treatment | VAS | Serious | Serious | Not serious | Serious | None | ⨁〇〇〇Very low |
| Cao 2010 | Herpes zoster | Wet cupping VS. medications | Number of patients with PHN after treatment | Serious | Not serious | Not serious | Serious | publication bias strongly suspected | ⨁〇〇〇Very low |
| Yuan 2015 | Chronic neck pain | Cupping VS. Waitlist | VAS | Not serious | Not serious | Not serious | Serious | None | ⨁⨁⨁〇Moderate |
|  | Chronic low back pain | Cupping VS. Medications | VAS | Not serious | Serious | Not serious | Not serious | None | ⨁⨁⨁〇Moderate |
| Shen 2022 | Low back pain | wet cupping VS. non-cupping group | VAS | Serious | Not serious | Not serious | Serious | None | ⨁⨁〇〇Low |
|  |  |  | PPI | Serious | Serious | Not serious | Serious | None | ⨁〇〇〇Very low |
| Xie 2022 | Nonspecific low back pain | blood pricking and cupping VS. other treatments | VAS | Not serious | Serious | Not serious | Not serious | None | ⨁⨁⨁〇Moderate |

MPPI=McGill present pain index; NPQ=Northwick Park Neck Pain Questionnaire; NRS: Numerical Rating Scale; PHN=Postherpetic neuralgia; PPI=present pain intensity; PPT=Pain pressure thresholds; SMPQ=Short Form McGill Pain Questionnaire; VAS=Visual analog scale; WOMAC = Western Ontario and McMaster Universities Osteoarthritis Index.
